# Fine mapping of a QTL affecting levels of skatole on pig chromosome 7

**DOI:** 10.1186/s12863-017-0549-8

**Published:** 2017-10-11

**Authors:** Maren van Son, Matthew P. Kent, Harald Grove, Rahul Agarwal, Hanne Hamland, Sigbjørn Lien, Eli Grindflek

**Affiliations:** 1 Topigs Norsvin, Storhamargata 44, 2317 Hamar, Norway; 2 Centre for Integrative Genetics (CIGENE), Department of Animal and Aquacultural Sciences, Faculty of Biosciences, Norwegian University of Life Sciences, P. O. Box 5003, 1432 Ås, Norway

**Keywords:** Boar taint, Skatole, Fine mapping, QTL, Whole genome re-sequencing, Pigs

## Abstract

**Background:**

Previous studies in the Norwegian pig breeds Landrace and Duroc have revealed a QTL for levels of skatole located in the region 74.7–80.5 Mb on SSC7. Skatole is one of the main components causing boar taint, which gives an undesirable smell and taste to the pig meat when heated. Surgical castration of boars is a common practice to reduce the risk of boar taint, however, a selection for boars genetically predisposed for low levels of taint would help eliminating the need for castration and be advantageous for both economic and welfare reasons. In order to identify the causal mutation(s) for the QTL and/or identify genetic markers for selection purposes we performed a fine mapping of the SSC7 skatole QTL region.

**Results:**

A dense set of markers on SSC7 was obtained by whole genome re-sequencing of 24 Norwegian Landrace and 23 Duroc boars. Subsets of 126 and 157 SNPs were used for association analyses in Landrace and Duroc, respectively. Significant single markers associated with skatole spanned a large 4.4 Mb region from 75.9–80.3 Mb in Landrace, with the highest test scores found in a region between the genes *NOVA1* and *TGM1* (*p* < 0.001). The same QTL was obtained in Duroc and, although less significant, with associated SNPs spanning a 1.2 Mb region from 78.9–80.1 Mb (*p* < 0.01). The highest test scores in Duroc were found in genes of the granzyme family (*GZMB* and *GZMH-like*) and *STXBP6*. Haplotypes associated with levels of skatole were identified in Landrace but not in Duroc, and a haplotype block was found to explain 2.3% of the phenotypic variation for skatole. The SNPs in this region were not associated with levels of sex steroids.

**Conclusions:**

Fine mapping of a QTL for skatole on SSC7 confirmed associations of this region with skatole levels in pigs. The QTL region was narrowed down to 4.4 Mb in Landrace and haplotypes explaining 2.3% of the phenotypic variance for skatole levels were identified. Results confirmed that sex steroids are not affected by this QTL region, making these markers attractive for selection against boar taint.

**Electronic supplementary material:**

The online version of this article (10.1186/s12863-017-0549-8) contains supplementary material, which is available to authorized users.

## Background

Boar taint is an unpleasant smell and/or taste of meat from some uncastrated male pigs. In most countries, the problem is solved by castrating piglets at a young age. Banning surgical castration would, however, be advantageous due to ethical and economic reasons, and the EU aims for alternative solutions to the boar taint issue within few years [1].

The two main compounds responsible for boar taint are androstenone and skatole (3-methylindole). Androstenone is a steroid hormone produced in the testicles, via the same biological pathway as testosterone and estrogens, whereas skatole is a fermentation product produced by degradation of tryptophan in the intestine [2, 3]. Both compounds are metabolized in the liver; however, deficient degradation leads to their accumulation in adipose tissue. Indole, another metabolite of tryptophan, also contributes to boar taint, but is less pronounced than the two other compounds [4, 5]. Levels of skatole and indole in adipose tissue are highly correlated [6–8], this is also true for androstenone and skatole [5, 7, 9]. Androstenone inhibits skatole metabolism [10–12], explaining why elevated levels of skatole is mainly a problem in male pigs. Heritabilities for androstenone and skatole in the range of 0.5–0.7 and ~0.4 have been reported for Norwegian Landrace and Duroc, respectively [7], suggesting that these boar taint compounds can be reduced by selective breeding. Our previous studies found very high genetic correlations of boar taint compounds to testosterone and estrogens (0.8–0.9 for androstenone and 0.4–0.6 for skatole, respectively) [7]. However, some studies have suggested that selection for low levels of boar taint should be feasible as they found no negative correlations between boar taint and male fertility [13] or production traits [14–17]. Other studies, on the other hand, show negative correlations between boar taint and male fertility [18, 19] and boar taint and meat quality [20]. Therefore, even though genetic selection is a promising alternative to reduce boar taint [13–16, 19, 21, 22], unfavorable correlations to steroid hormones as well as uncertainty on the cost/benefit of including boar taint in the breeding goal [1] have slowed down practical implementation in breeding. Thus, genetic markers breaking unfavorable correlations between boar taint compounds and sex steroids may be potent selection candidates.

Several QTL studies have been conducted to reveal genomic regions underlying boar taint [7, 23–32]. Results are quite inconsistent but with different breeds, age of the boars and definition/measurements of the traits partly explaining why results differ between studies. The region on SSC7, however, seems to consistently affect both androstenone and skatole levels in Norwegian Landrace and Duroc [7, 31] as well as in other breeds [23–25, 29]. The cytochrome P450 members *CYP1A1* and *CYP1A2* have been suggested as candidate genes for skatole and are located on SSC7 [31, 33]. No functional mutations have, however, been detected in any of the studies. Also, the genomic region obtained in the Norwegian populations [31] seems to include three different QTLs for androstenone and two for skatole.

The aim of this study was to fine map the most significant region affecting the level of skatole, found on SSC7 at 74.7–80.5 Mb (*Sus scrofa* build10.2 positions) [31]. This QTL was identified by LDLA analysis and did not affect levels of testosterone or estrogens, making it particularly interesting for implementation in breeding. To assess the role of this QTL in levels of skatole, we performed fine mapping of the region by selecting SNPs from whole genome re-sequencing data, followed up by genotyping and association analyses.

## Results

Whole genome re-sequencing was performed on 24 Landrace and 23 Duroc pigs, and provided a total of 10.1 billion paired-end reads (PE; 2 × 100 base pairs) with a per-animal genome coverage ranging from 9-17X. Initial quality control removed approximately 15% of the reads and the remaining reads were mapped against the pig reference genome (*Sscrofa 10.2*) with an overall mapping percentage of 77%. SNP detection was performed in a 5.8 Mb QTL region for skatole on SSC7 (74.7–80.5 Mb). After filtering, 3836 SNPs were found in common for Landrace and Duroc for the QTL region, and 166 of these were selected for genotyping. Additionally, 22 and 23 SNPs from the Illumina 60 K BeadChip were available in the QTL region for the Landrace and Duroc boars, respectively. SNP filtering on minor allele frequency (MAF) and call rate made 126 and 157 SNPs available for association analyses in Landrace and Duroc, respectively.

Association analyses was performed to identify SNPs and haplotypes associated with skatole in the SSC7 QTL region. The SNPs were also tested for association to levels of indole, androstenone, testosterone, estradiol and estrone sulphate [See Additional files 1 and 2 for results for Landrace and Duroc, respectively]. To determine if the region contains more than one QTL we reanalyzed the data with the most significant SNP included as a fixed effect and checked if this influenced the test scores of the other markers in the region. The test revealed no other significant SNPs in the region, suggesting that the associations are caused by one QTL only.

**Fig. 1:**
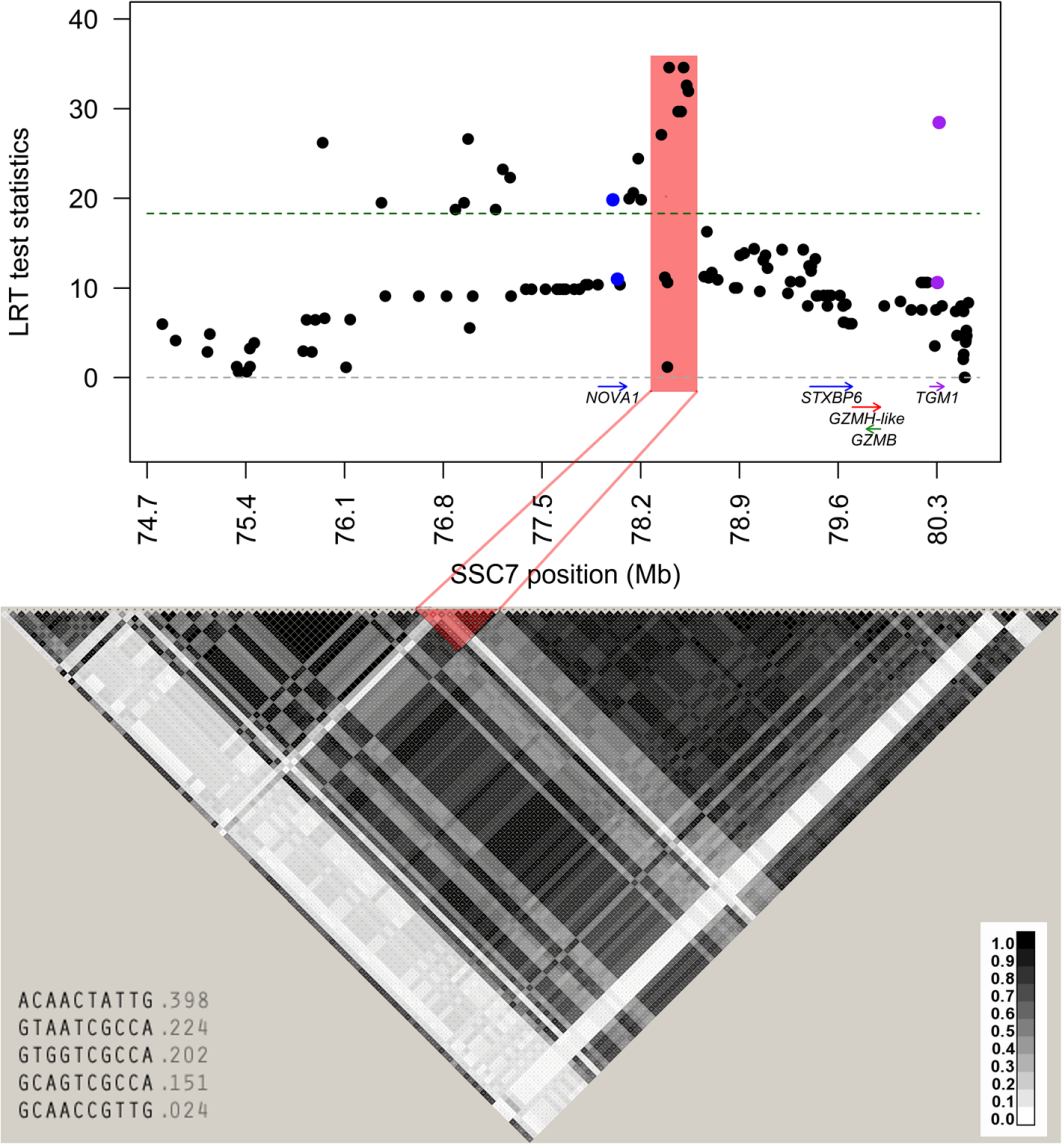
Region plot of the single marker associations between SSC7 SNPs and skatole in Landrace. Test statistics (LogLikelihood (LRT) scores) is shown along the y axis and physical position in MegaBases (Mb) is shown along the x axis, together with genes holding significant SNPs. An additional file show the complete list of genes in the region [see Additional file 3]. The threshold of significance is indicated as a dashed line (multiple testing adjusted *p* = 0.001). Linkage Disequilibrium (r^2^) between SNPs as estimated by Haploview is plotted below and the haplotype block is indicated in red. The darker shades of black represent stronger r^2^. Haplotypes in the block and their frequencies are presented below the LD plot

The highest log likelihood-ratio test scores (LRT) for skatole in Landrace were found for SNPs *rs321605443* and *rs330435414* at 78.4 and 78.5 Mb, respectively (LRT = 34.6; explaining 5.0% of the phenotypic variation). These SNPs are located in an intergenic region between a 5S rRNA gene *(ENSSSCG00000018509)* and *syntaxin binding protein 6 (amisyn) (STXBP6)* with extensive LD (Fig. 1). Altogether, 21 SNPs were significantly associated with skatole using LRT values corrected for multiple testing (*p* < 0.001) in the region 75.9–80.3 Mb.

**Fig. 2:**
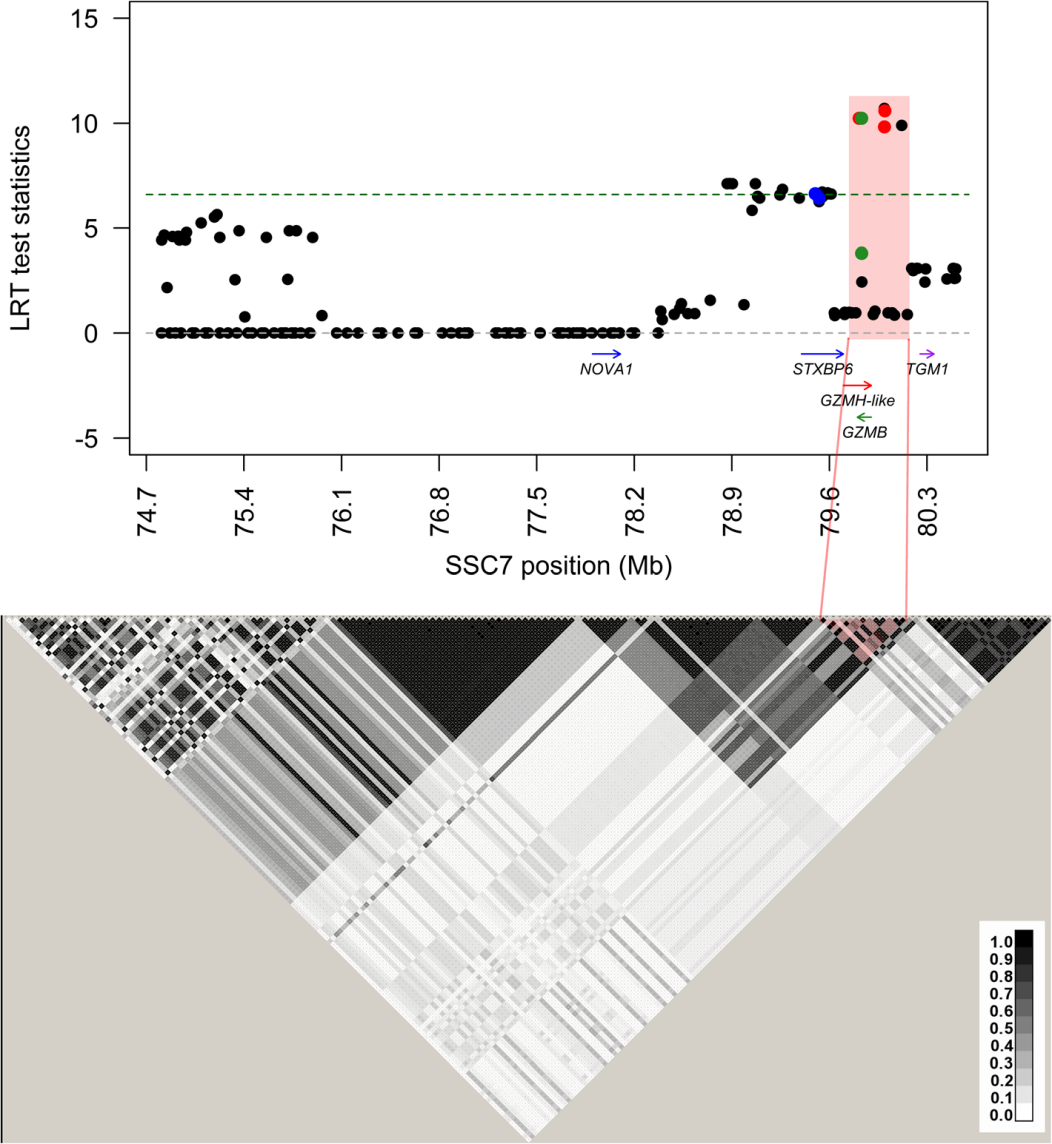
Region plot of the single marker associations between SSC7 SNPs and skatole in Duroc. Test statistics (LogLikelihood (LRT) scores) is shown along the y axis and physical position in MegaBases (Mb) is shown along the x axis together with genes holding significant SNPs. A complete list of genes in the region can be found in Additional file 3. The threshold of significance is indicated as a dashed line (*p* = 0.01). Linkage Disequilibrium (r^2^) between SNPs as estimated by Haploview is plotted below and haplotype block is indicated in red. The darker shades of black represent stronger r^2^

The most significant results on SSC7 in Duroc was found for SNP *rs431825253* at 80 Mb (LRT = 10.7; explaining 2.6% of the phenotypic variation). When considering a *p*-value of 0.01 (LRT > 6.6), 18 SNPs in the 78.9–80.1 Mb region were found significant in the current study. The highest test scores in Duroc were found in *GZMB* (*granzyme B (granzyme 2, cytotoxic T-lymphocyte-associated serine esterase 1)*)*, GZMH-like* and *STXBP6* (Fig. 2).

The most significant SNPs in Landrace, located between 78.3–78.5 Mb were grouped into a haplotype block of ten SNPs (Fig. 1). Association analyses revealed that haplotypes within this block are significantly associated with levels of skatole (LRT = 24.3) and that they explain 2.3% of the phenotypic variation. A haplotype block was constructed for the most significant SNPs in Duroc at 79.8–80.1 Mb (Fig. 2). Haplotypes within this block was not significantly associated with skatole (LRT = 3.2).

## Discussion

Skatole is a major contributor to boar taint and selection against this compound would be advantageous for both economic and ethical reasons. The correlation between skatole and sex steroids is lower that the correlation between androstenone and sex steroids [7]. This makes selection against high levels of skatole, as compared to androstenone, less likely to affect reproduction in the pigs.

### Fine mapping in Landrace

Only two of the 21 significant SNPs are located inside genes, one in the first intron of *Neuro-oncological ventral antigen 1* (*NOVA1*) and one in the 3’UTR region of *Transglutaminase 1* (*TGM1*). The other 19 significant SNPs were located in a 2.5 Mb segment in the intergenic regions *NOVA1 - 5S rRNA (ENSSSCG00000018509), ENSSSC00000024192* -*ENSSSC00000022792* and *5S rRNA (ENSSSCG00000018509) - STXBP6*.

NOVA1 is a protein that controls alternative splicing of mRNAs in different cell types [34–36], whereas TGM1 is a membrane-bound enzyme that helps to protect against infections and water loss [37] and is associated with skin diseases [38]. To our current knowledge, these genes do not seem to have any relevance to skatole and we find it more likely that other mechanisms or genes in region, whose function is not yet characterized in pigs, are involved [for a list of all genes in the region see Additional file 3]. Intergenic SNPs may affect distal regulatory regions of candidate genes by changing transcription factor binding sites, interfering with chromatin signaling or by bringing chromosomes together in the nucleus [39]. Evolutionary conserved non-coding regions are more likely to contain regulatory motifs [39]. Some of the most significant SNPs in Landrace (*rs322137732, rs344485447, rs326608782, rs431825241, rs321605443, rs344465955, rs330435414, rs334377914* with LRT scores of 19.8–34.6) showed 65–82% sequence similarity to human *NOVA1* - *STXBP6* intergenic regions when sequences of 200–400 bp surrounding the SNPs were blasted. However, no sequence hits were found for other mammals besides human, making it difficult to predict the degree of evolutionary conservation in this region.

Sequence surrounding *rs321711075* (LRT = 20.9) showed a 68% sequence similarity to the human long non-coding (lncRNA) RNA LINC00645, and the regulatory properties of lncRNA may give new meaning to GWAS associations in non-coding genomic regions [40]. It has been shown that lncRNAs are abundant in gene deserts associated with genetic traits in human [41], and there are examples where specific intergenic lncRNAs are involved in trait regulation through chromatin modifications (e.g. [42, 43]). Because there are no known sequence motifs common to long non-coding RNAs they are difficult to annotate [44] and the search for lncRNAs in the pig genome has just begun [45]. Whether the QTL signal for skatole in Landrace is caused by regulatory activity of a lncRNA needs to be further investigated.

None of the SNPs investigated were associated with levels of indole, androstenone, testosterone, estradiol and estrone sulphate, confirming the results of Grindflek et al. 2011 [31]. This makes the QTL particularly interesting for breeding purposes, as it would not influence the levels of sex steroids.

### Fine mapping in Duroc

The results in Duroc were less significant than those found in Landrace, which is in agreement with our earlier QTL study [31]. The QTL peak in Duroc was in a slightly different chromosomal position than the most significant SNPs in Landrace. This may indicate that different genetic mechanisms are contributing to this QTL in the two breeds, but might also be due to differences in allele frequencies and LD structure. Furthermore, the average skatole levels are lower in Duroc (0.06 μg/g) than in Landrace (0.10 μg/g), which possibly also affects our results. The most significant SNPs associated with skatole, located in genes belonging to the granzyme family, were associated with levels of indole and androstenone in fat. The alleles associated with low levels of skatole were also associated with low levels of androstenone, indicating that selection for low boar taint is possible. No associations were found for levels of testosterone, estradiol or estrone sulphate.

The most significant results for skatole levels were found in a gene dense region on SSC7 where seven of the 18 significant SNPs are in genes of the granzyme family: *GZMB* and *GZMH-like*, while four are in the gene *STXBP6* (Fig. 2). One significant SNP is non-synonymous, but predictions using SIFT [46] do not suggest any change in protein function. Granzymes are serine proteases found in immune related cell types such as cytotoxic T lymphocytes and natural killer cells where they play a role in eliminating diseased cells [47]. Interestingly, tryptophan catabolism is also involved in regulation of immune responses [48]. Specifically, granzyme B induction by interleukin has been associated with up-regulation of other immunoregulatory proteins including indoleamine 2,3-dioxygenase (IDO) [49] which is the rate-limiting enzyme in tryptophan metabolism [50]. Although the connection between granzymes and skatole is not straight forward, different levels of tryptophan could reflect levels of skatole and thereby explain our results.

Two significant SNPs are located in introns of *STXBP6*. STXBP6 binds to the SNARE complex [51] and is involved in vesicle-mediated transport. The gene *STX5A*, encoding another syntaxin involved in SNARE interactions and vesicular transport, has been found down-regulated in rats by treatment of indole-3-carbinol, one of the metabolites of skatole [52]. If skatole metabolites also have an effect on syntaxin genes in pigs, it might explain our results, but further studies are required to clarify any effect of this kind.

### Haplotype associations

The haplotype block in Landrace showed significant association to levels of skatole. The most frequent haplotype in the block (frequency ~0.4) was associated with lower levels of skatole whereas the second most frequent haplotype (frequency ~0.22) was associated with higher levels. The difference in mean skatole levels between animals homozygous for these two haplotypes was 0.06 μg/g skatole. Considering consumer acceptance levels of 0.2 μg/g, 14.5% of Norwegian Landrace boars have been shown to have skatole levels that are too high [7]. The SNPs within these haplotype blocks can therefore be used as genetic markers for lowering the overall skatole levels in this Landrace population.

## Conclusions

Fine mapping of a QTL for skatole on SSC7 was conducted to narrow down the QTL region and search for genes and mechanisms underlying the QTL. The most significant results were found in the intergenic regions between 75.9–78.5 Mb and in *NOVA1* and *TGM1* in Landrace. In Duroc, SNPs within *GZMB, GZMH-like, STXBP6* and intergenic regions at 78.8–80.1 Mb were found to be most significant. The region in Duroc was also associated with levels of indole and androstenone. Although no causal variant was detected, genetic markers for boar taint that are not associated with sex steroids have been identified and would therefore be highly relevant for selection purposes.

## Methods

### Animals and phenotypes

For re-sequencing purposes, 23 Norwegian Duroc and 24 Norwegian Landrace boars used in the Norsvin breeding program from 2010 to 2013 were selected. The boars were key individuals in our previous QTL studies, with either high or low levels of boar taint [7, 31], or frequently used AI boars during these three years, in order to catch as much as possible of the genetic variation present in the population.

A total of 911 Duroc and 767 Landrace boars from Norsvin’s boar testing station were genotyped in this study. The boars include fathers and sons from 70 Duroc and 92 Landrace half sib families, and 60 K BeadChip genotypes (Illumina) were available for all the boars. For Landrace, another 440 sons were available with 60 K genotypes from the previous study [31], making imputation to the new markers genotyped in this study feasible, and these boars were also included in the association analysis. For Duroc, all the available boars were genotyped in this study. Animals were reared under similar conditions using standard commercial feed and were sacrificed over a period of 26 months. On average, the Duroc and Landrace boars reached slaughter weight (100 kg) at 156 and 143 days, respectively, and were slaughtered on average 15 days later. Blood samples for DNA extraction were collected before slaughter and subcutaneous adipose tissue samples from the neck for skatole measurements were collected at the slaughter line and stored at −40 °C until chemical analyses were performed. The boars were slaughtered in compliance with national guidelines. The pigs were stunned in an atmosphere with 90% CO_2_, and the carcasses were ex-sanguinated, scalded and split within 30 min post mortem.

Levels of skatole were measured in subcutaneous fat, at the Hormone laboratory, NMBU, using high performance liquid chromatography [8] whereas levels of androstenone in fat and plasma were analyzed by a modified time-resolved fluoroimmunoassay [53] and using antibody by Andresen [54]. Plasma levels of testosterone, estradiol and estrone sulphate were analyzed at the Hormone laboratory at Oslo University Hospital. The plasma levels of testosterone were measured by a radioimmunoassay (Orion Diagnostica, Espoo, Finland) whereas plasma levels of 17β-estradiol were measured by a fluoroimmunoassay (Perkin Elmer, Turku, Finland). Levels of estrone sulphate in plasma were measured by a radioimmunoassay (Diagnostic System Laboratories, Inc., Webster, TX, USA). More information about chemical analyses and compound levels can be found in Grindflek et al. [7].

DNA for genotyping purposes was extracted from blood using the MagAttract DNA Blood Midi M48 protocol on the Bio-Robot M48 (Qiagen, Hilden, Germany). DNA concentration and quality were evaluated using a NanoDrop ND-1000 spectrophotometer (NanoDrop Technologies, DE, USA).

### Re-sequencing, pre-processing and read mapping

Genomic DNA from 23 Duroc and 24 Landrace boars sequenced on an Illumina GAII (Illumina, San Diego, USA). The sequencing was performed by a commercial sequencing center (Fasteris, Switzerland) according to manufacturer’s protocols. FastQC version 0.10.1 (Babraham Bioinformatics, UK) was used for quality checking, revealing an overall per-base quality ≥30. Pre-processing of reads was done using a custom Perl script written to remove duplicated reads, trim sequencing primer sequence and remove reads shorter than 0.8 of their original length. On average, 15% of reads were filtered using this pipeline and the remaining reads were aligned to the *Sus scrofa* 10.2 reference genome [55] using the software Bowtie2 version 2.0.0 with default parameters [56]. Mapped reads were sorted by their chromosomal coordinates using Samtools version 0.1.18 [57]. The Picard *AddOrReplaceReadGroups* program (http://broadinstitute.github.io/picard/) was used to assign unique IDs to the files before SNP calling.

### SNP detection, annotation and selection

SNPs were detected within each breed using Freebayes [58] generating a list of SNPs within the QTL region on SSC7 where each SNP was supported by a minimum of two reads across the samples. SNPs located within repeat regions (as defined by pig ensembl release 67) were removed. Moreover, to reduce the chances of false positive SNPs, the following two filtering criteria were applied: 1) The minimum total coverage of the reference allele was two, and 2) both the two homozygous and the heterozygote genotype had to be present for each SNP. The read depths for SNP positions across all sequenced samples were in the range of 12 to 882, with a mean count of 506, whereas the MAF of the SNPs ranged from 0.03 to 0.5 with a mean count of 0.22. After initial filtering a list of common SNPs for Duroc and Landrace was made, comprising 3836 SNPs within this region. The Variant Effect Predictor (VEP) [59] was used to annotate SNPs to gene structure elements (including exons, introns, UTRs) and to classify variants (e.g. missense, nonsense, synonymous, stop gain/loss). SNPs were selected for genotyping based on their position in order to cover the whole QTL region. Furthermore, non-synonymous SNPs were prioritized. A total of 166 SNPs were selected for genotyping. The SNPs have been submitted to NCBI dbSNP [60]. Sequences around some of the identified SNPs were searched for similarity against human using BLAST [61].

### Genotypes and phase inference

The boars were genotyped using matrix-assisted laser desorption/ionization time-of-flight mass spectroscopy (MALDI-TOF MS) assays. Assays were designed using MassARRAY Assay Design software (Agena Biosciences, Hamburg, Germany) at multiplexing levels between 8 and 27 [See Additional file 4 for assay primers], and genotyping was done using the IPLEX protocol according to the manufacturer’s instructions. Genotypes were also retrieved for porcine 60 K Illumina BeadChip SNPs in this QTL region for the same boars, as available from Grindflek et al. 2011 [31]. SNP filtering was done for minor allele frequency (>0.05) and call rate (>0.95), reducing the number of SNPs to 157 and 126 for Duroc and Landrace, respectively, in the QTL region. The BEAGLE software version 1.0.0 [62] was used to phase chromosomes and impute sporadic missing genotypes. The Haploview software version 4.2 [63] was applied to calculate pair-wise LD measures for all SNP pairs and the “four gamete rule” method, as implemented in Haploview, was used to define haplotype blocks.

### Single marker and haplotype association analysis

Association analysis was done for skatole, indole, androstenone, testosterone, 17β-estradiol and estrone sulphate levels for all SNPs using the mixed model:

- y = sire + herd-year-season + wait-station + pen + animal + sample-date + SNP/haplotype + age_25kg + days-test + days-wait + liveborn + (liveborn)^2^ + e

Here, y is the phenotype expressed as ln(μg/g levels of the phenotype) and the fixed effects are sire, herd-year-season, waiting in boar test station before slaughter or not, and pen. Covariates used were age at 25 kg (start of boar test), age from 25 kg to 100 kg (days in boar test), days from 100 kg to slaughter (days in waiting station) and number of live born in same litter. Animal ID, sample date for adipose tissue and SNP/haplotype were fitted as random effects. To test if the QTL region contained more than one QTL, the dataset was re-analyzed fitting the most significant SNP as a fixed effect in the model above.

Association mapping was conducted using the ASReml software v.2.00 [64]. Log likelihood (LogL) ratios for each SNP or haplotype were estimated as the difference in LogL value between models with and without this effect. Log-likelihood ratio test (LRT) scores were calculated as two times the (LogL) and LRT was assumed approximately chi-square distributed with one degree of freedom. Multiple testing correction was done to adjust the significance threshold with the effective number of independent tests (MeffG) [65].

## Additional files

Additional file 1: Summary of LogLikelihood test scores (LRT values) for all SNPs and compounds examined in Landrace. SNPs are presented with their IDs (rs# or 60 K ID), position on *Sscrofa10.2*, functional classes and their effect, amino acid change, SIFT prediction and LRT scores for skatole, indole, androstenone in fat and plasma, testosterone, estradiol and estrone sulphate. (XLSX 18 kb)

Additional file 2: Summary of LogLikelihood test scores (LRT values) for all SNPs and compounds examined in Duroc. SNPs are presented with their IDs (rs# or 60 K ID), position on *Sscrofa10.2*, functional classes and their effect, amino acid change, SIFT prediction and LRT scores for skatole, indole, androstenone in fat and plasma, testosterone, estradiol and estrone sulphate. (XLSX 22 kb)

Additional file 3: All genes in the 5.8 Mb SSC7 QTL region (74.7–80.5 Mb, build 10.2) according to Ensembl. (XLSX 23 kb)

Additional file 4: Primers used for genotyping. (XLSX 26 kb)
